
JOURNAL INFORMATION
==============================
NLM Title Abbreviation: CPT Pharmacometrics Syst Pharmacol
Journal ID: CPT Pharmacometrics Syst Pharmacol
NLM Journal ID: 101580011
Journal ID: PSP4

CPT: Pharmacometrics & Systems Pharmacology

EISSN: 2163-8306
Publisher: Wiley

ARTICLE INFORMATION
==============================
PMCID: PMC11474608
DOI: 10.1002/psp4.12827
Article ID: PSP412827
Article version: 1
Subjects: Issue Information, Issue Information

Issue Information

Electronic publication date: 2023 Dec 16
Publication date: 2023 Dec
Volume: 12
Issue: 12
First page: 1819
Last page: 1822
Copyright: © 2023 ASCPT
License: This is an open access article under the terms of the http://creativecommons.org/licenses/by/4.0/ License, which permits use, distribution and reproduction in any medium, provided the original work is properly cited.
License URL: https://creativecommons.org/licenses/by/4.0/

Figure count: 0
Table count: 0
Page count: 3

source-schema-version-number: 2.0
cover-date: December 2023
details-of-publishers-convertor: Converter:WILEY_ML3GV2_TO_JATSPMC version:6.4.9 mode:remove_FC converted:15.10.2024

==============================

An Official Journal of ASCPT and ISoP

### CPT: Pharmacometrics & Systems Pharmacology

Open Access

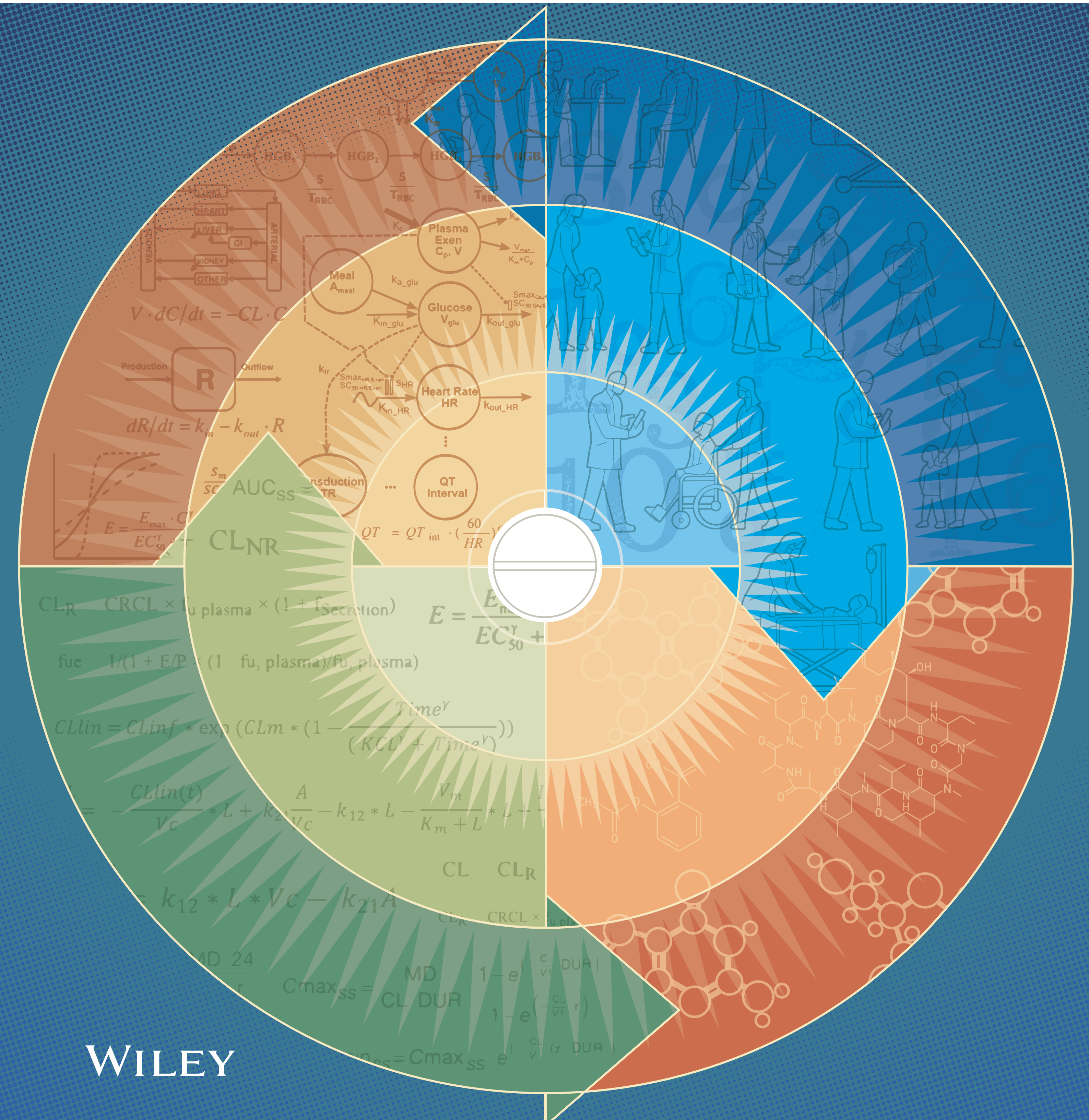

WILEY

### CPT: Pharmacometrics & Systems Pharmacology

WILEY

### Editor-in-Chief

France Mentré, MD, PhD, *School of Medicine of University Paris Diderot, Paris, France*

### Deputy Editor-in-Chief

Karen Rowland Yeo, PhD, *Certara UK Ltd., Sheffield, UK*

### Associate Editors

Paolo Denti, PhD, *University of Cape Town, Cape Town, South Africa*
Jonathan French, ScD, *Metrum Research Group, Tariffville, CT, USA*
Stefanie Hennig, MSc, PhD, *Certara Strategic Consulting, Brisbane, Australia*
Priya Jayachandran, PharmD, *Regeneron Pharmaceuticals, Inc., San Francisco, CA, USA*
Andrew Hooker, PhD, *Uppsala University, Uppsala, Sweden*
Sriram Krishnaswami, PhD, *Pfizer, Groton, CT, USA*
Ping Zhao, PhD, *Bill & Melinda Gates Foundation, Seattle, WA, USA*

### Editors-in-Training

Brian Cicali, PhD, *University of Florida, College of Pharmacy, Orlando, FL, USA*
Jane Knöchel, PhD, *AstraZeneca , Mölndal, Sweden*

### American Society for Clinical Pharmacology and Therapeutics

***Chief Executive Officer***
Abigail W. Gorman, *Alexandria, VA*

***Chief Operating Officer***
Elise S. Laffman-Johnson, *Alexandria, VA*

***Managing Editor***
Alethea B. Gerding, *Alexandria, VA*

***Deputy Managing Editor***
Alaina Webster, *Alexandria, VA*

***Senior Editorial Coordinator***
Aimee Greene, *Alexandria, VA*

### Editorial Board

Vivaswath S. Ayyar, PhD, *Janssen Research & Development, Spring House, PA*
Robert Bies, PharmD, PhD, *State University of New York at Buffalo, Buffalo, NY, USA*
Marc Birtwistle, PhD, *Clemson University Clemson, SC, USA*
Yuan Chen, PhD, *Genentech South San Francisco, CA, USA*
Marylore Chenel, PharmD, PhD, *Pharmetheus Uppsala, Sweden*
Emmanuel Chigutsa, PhD, *Eli Lilly and Company, Indianapolis, IN, USA*
Teresa Dalla Costa, PhD, *Federal University of Rio Grande do Sul - UFRGS Porto Alegre, Brazil*
Rajat Desikan, PhD, *GSK, UK, Middlesex, UK*
Narendra M Dixit, PhD, *Indian Institute of Science, Bengaluru, India*
Anne-Gaelle Dosne, PharmD, PhD, *Janssen Research & Development, Beerse, Belgium*
Thomas Dumortier, Eng, MSc, *Novartis Pharma AG, Basel, Switzerland*
James M. Gallo, PharmD, PhD, *University at Buffalo, Buffalo, NY, USA*
Wei Gao, PhD, *EMD Serono, Billerica, MA, USA*
Jérémie Guedj, PhD, *INSERM, Paris, France*
Manish Gupta, PhD, FCP, *GenMab; Princeton, NJ, USA*
Bart Hendriks, PhD, *Novartis Institutes for BioMedical Research Cambridge, MA, USA*
Nick Holford, MBChB, *The University of Auckland Auckland, New Zealand*
Brett A. Howell, PhD, *DILIsym Services Division, Simulations Plus, Inc., Research Triangle Park, NC, USA*
Manuel Ibarra, PhD, *Universidad de la República, Montevideo, Uruguay*
Siv Jönsson, PhD, *Uppsala University Sweden, Uppsala, Sweden*
Kristin Karlsson, PhD, *Uppsala University Uppsala, Sweden*
Helen Kastrissios, PhD, *QuanTx Consulting, Brisbane, Australia*
Ron Keizer, PhD, *InsightRX San Francisco, CA, USA*
Steven E. Kern, PhD, *Global Health Labs, Bellevue, WA, USA*
Akash Khandelwal PhD, *UCB Biosciences GmbH, Monheim am Rhein, Germany*
Andrzej Kierzek, PhD, *Certara QSP; Sheffield, United Kingdom*
Qi Liu, PhD, *US Food and Drug Administration, Silver Spring, MD*
James Lu, PhD, *Genentech, Inc., South San Francisco, CA, USA*
Rajanikanth (Raj) Madabushi, PhD, *US Food and Drug Administration, Silver Spring, MD*
Efthymios Manolis, MSc, *European Medicines Agency London, United Kingdom*
Samer Mouksassi, PharmD, PhD, *Certara, Cairo, Egypt*
Jackson K. Mukonzo, PhD, *Makerere University, Kampala Uganda*
Joakim Nyberg, PhD, *Pharmetheus, Uppsala, Sweden*
Elsje Pienaar, PhD, *Weldon School of Biomedical Engineering, Purdue University, West Lafayette, IN, USA*
Daniel Scholes Rosenbloom, PhD, *Merck & Co., Rahway, NJ, USA*
Joe Standing, PhD, *University College London London, United Kingdom*
Natasha Strydom, PhD, *Amgen, Inc., San Francisco, CA, USA*
Yuichi Sugiyama, PhD, *Riken Innovation Center Sugiyama Laboratory Yokohama, Japan*
Elin Svensson, PhD, *Uppsala University, Uppsala, Sweden*
Joel Tarning, PhD, *Mahidol-Oxford Tropical Medicine Research Unit Bangkok, Thailand*
Nadia Terranova, PhD, *Merck KGaA, Lausanne, Switzerland*
Michael Tortorici, PharmD, PhD, *Aro Biotherapeutics, Philadelphia, PA, USA*
Coen van Hasselt, PhD, *Leiden University, Leiden, the Netherlands*
Paolo Vicini, PhD, MBA, *Confo Therapeutics, Ghent, Belgium*
Camille Vong, PhD, *Novartis AG Pharma, Basel, Switzerland*
Xinyuan (Susie) Zhang, PhD, *Daiichi Sankyo, Inc. Basking Ridge, NJ, USA*

### Overview

*CPT: Pharmacometrics & Systems Pharmacology* (PSP) is an official open access journal of the American Society for Clinical Pharmacology and Therapeutics (ASCPT) and International Society of Pharmacometrics (ISoP).

*CPT: Pharmacometrics & Systems Pharmacology* is a cross-disciplinary journal devoted to publishing advances in quantitative (e.g., modeling and simulation) methods as applied in pharmacology, physiology and therapeutics in humans. The journal welcomes original research articles, reviews and tutorials that bridge the following areas: pharmacometrics, modeling and simulation as applied to the design and evaluation of clinical trials, systems pharmacology modeling, particularly with a mechanistic link to human (patho)physiology, disease modeling, “population” or mixed-effects pharmacokinetics and pharmacodynamics (PKPD) modeling, modeling and simulation to support translational research, physiologically-based pharmacokinetics (PBPK), model-based meta-analyses of clinical trials, mechanism-based pharmacokinetic-pharmacodynamic modeling, computational pharmacology, bioinformatics, comparative efficacy, effectiveness and cost-effectiveness. Systems pharmacology may involve the application of systems biology approaches to study drug activities, targets and effects. The discipline is often defined with reference to engineering and pharmacological principles as the quantitative analysis of the dynamic interactions between drugs and a biologic system that aims to understand the behavior of the system as a whole. The common focus will be on quantitative methods that improve our understanding of pharmacology and therapeutics in humans.

**Abstracting and Indexing**
- Embase (Elsevier)
- SCOPUS (Elsevier)
- MEDLINE/PubMed indexing

**Society Information**
The American Society for Clinical Pharmacology and Therapeutics (ASCPT) was founded in 1900, and consists of over 2,200 professionals whose primary interest is to advance the science and practice of clinical pharmacology and translational medicine for the therapeutic benefit of patients and society. ASCPT is the largest scientific and professional organization serving the disciplines of Clinical Pharmacology and Translational Medicine.

**All articles published in this journal are freely available to read, download, and share. Articles are published under the terms of the Creative Commons Attribution Non-Commercial License which permits use, distribution, and reproduction in any medium, provided the original work is properly cited and is not used for commercial purposes.**

**Disclaimer**
The Publisher, the American Society for Clinical Pharmacology and Therapeutics, the International Society of Pharmacometrics, and Editors cannot be held responsible for errors or any consequences arising from the use of information contained in this journal; the views and opinions expressed do not necessarily reflect those of the Publisher, the American Society for Clinical Pharmacology and Therapeutics, the International Society of Pharmacometrics or Editors, neither does the publication of advertisements constitute any endorsement by the Publisher, the American Society for Clinical Pharmacology and Therapeutics, the International Society of Pharmacometrics or Editors of the products advertised.

**Publisher**
*CPT: Pharmacometrics & Systems Pharmacology* is published by Wiley Periodicals, LLC, 101 Station Landing, Suite 300, Medford, MA 02155; Telephone: 781-388-8200; Fax: 781-388-8210.

### CONTENTS

CPT: Pharmacometrics & Systems Pharmacology

### PERSPECTIVE

PERSPECTIVE

1823 Quantitative systems pharmacology in the age of artificial intelligence
**Benjamin Ribba**

### TUTORIAL

TUTORIAL

1827 Tutorial on model selection and validation of model input into precision dosing software for model-informed precision dosing
**Zachary L. Taylor, Ethan A. Poweleit, Kelli Paice, Katherine M. Somers, Kathryn Pavia, Alexander A. Vinks, Nieko Punt, Tomoyuki Mizuno and Sonya Tang Girdwood**

### RESEARCH

ARTICLES

1846 Model-based simulation to support the approval of isatuximab alone or with dexamethasone for the treatment of relapsed/refractory multiple myeloma in Japanese patients
**Hoai-Thu Thai, Kimiko Koiwai, Yoshihisa Shitara, Hirotaka Kazama, Jean-Baptiste Fau, Dorothée Semiond and Christine Veyrat-Follet**

1859 Population pharmacokinetics of molnupiravir in adults with COVID-19: Lack of clinically important exposure variation across individuals
**Sébastien Bihorel, Youfang Cao, Akshita Chawla, Ruthie Birger, Brian M. Maas, Wei Gao, Stefan Roepcke, Susanne Sardella, Rebecca Humphrey, Sindhuri Kondragunta, Bhuvana Jayaraman, Monika Martinho, Wendy Painter, George Painter, Wayne Holman, Carisa De Anda, Michelle L. Brown, Matthew G. Johnson, Amanda Paschke, Matthew L. Rizk and Julie A. Stone**

1872 Apixaban and rivaroxaban's physiologically-based pharmacokinetic model validation in hospitalized patients: A first step for larger use of a priori modeling approach at bed side
**Jean Terrier, Frédéric Gaspar, Pauline Gosselin, Olivier Raboud, Camille Lenoir, Victoria Rollason, Chantal Csajka, Caroline Samer, Pierre Fontana, Youssef Daali and Jean-Luc Reny for the OptimAT study group**

1884 Population pharmacokinetics of ivermectin after mass drug administration in lymphatic filariasis endemic communities of Tanzania
**Adam M. Fimbo, Eulambius M. Mlugu, Eliford Ngaimisi Kitabi, Gerald S. Kulwa, Mohammed A. Iwodyah, Rajabu Hussein Mnkugwe, Peter P. Kunambi, Alpha Malishee, Appolinary A. R. Kamuhabwa, Omary M. Minzi and Eleni Aklillu**

1897 Dosing recommendation of nirmatrelvir/ritonavir using an integrated population pharmacokinetic analysis
**Phylinda L. S. Chan, Ravi Shankar P. Singh, Donna S. Cox, Haihong Shi, Bharat Damle and Timothy Nicholas**

1911 Mid-dosing interval concentration is important for polymyxin B exposure and acute kidney injury in critically ill patients
**Jing Wang, Yuanchen Li, Siqi Huang, Min Wang, Lu Jin, Xuemei Luo, Xiaoliang Cheng, Na Yang and Huaijun Zhu**

1922 A multicompartment population PK model to predict tenofovir and emtricitabine mucosal tissue concentrations for HIV prevention
**Erick Leung, Mackenzie L. Cottrell, Craig Sykes, Nicole White, Angela D. M. Kashuba and Julie B. Dumond**

1931 Physiologically-based pharmacokinetic model to predict doxorubicin and paclitaxel exposure in infants through breast milk
**David Damoiseaux, Frédéric Amant, Jos H. Beijnen, Shelby Barnett, Gareth J. Veal, Alwin D. R. Huitema and Thomas P. C. Dorlo**

1945 Combining pharmacometric models with predictive and prognostic biomarkers for precision therapy in Crohn's disease: A case study of brazikumab
**Nan Zhang, Ming Liang Chan, Jing Li, Philip Z. Brohawn, Bo Sun, Inna Vainshtein, Lorin K. Roskos, Raffaella Faggioni and Rada M. Savic**

1960 Physiologically based mechanistic insight into differential risk of valproate hepatotoxicity between children and adults: A focus on ontogeny impact
**Yu-Ting Huang, Yen-Ming Huang, Fan-Lu Kung, Chun-Jung Lin, Tun Jao and Yunn-Fang Ho**

1972 Model-based assessment of neutrophil-mediated phagocytosis and digestion of bacteria across in vitro and in vivo studies
**Anders Thorsted, Anh Duc Pham, Lena E. Friberg and Elisabet I. Nielsen**

1988 Evaluation of the effect of erenumab on migraine-specific questionnaire in patients with chronic and episodic migraine
**Po-Wei Chen, Mats O. Karlsson, Sebastian Ueckert, Ari Pritchard-Bell, Cheng-Pang Hsu, Sandeep Dutta and Malidi Ahamadi**

2001 Case–control matching-guided exposure-efficacy relationship for avelumab in patients with urothelial carcinoma
**Pooneh Soltantabar, Ali Alhadab, Jennifer Hibma, Satrajit Roychoudhury, Diane D. Wang, Carlo Bello and Mohamed Elmeliegy**

2013 Population PK/PD modeling of low-density lipoprotein cholesterol response in hypercholesterolemic participants following administration of bococizumab, a potent anti-PCSK9 monoclonal antibody
**Ellen Q. Wang, Nitin Kaila, David Plowchalk, Leonid Gibiansky, Carla Yunis and Kevin Sweeney**

2027 Association between SARS-CoV-2 viral kinetics and clinical score evolution in hospitalized patients
**Nadège Néant, Guillaume Lingas, Alexandre Gaymard, Drifa Belhadi, Maya Hites, Thérèse Staub, Richard Greil, Jose-Artur Paiva, Julien Poissy, Nathan Peiffer-Smadja, Dominique Costagliola, Yazdan Yazdanpanah, Maude Bouscambert-Duchamp, Amandine Gagneux-Brunon, Florence Ader, France Mentré, Florent Wallet, Charles Burdet, Jérémie Guedj and the DisCoVeRy study group**

2038 A Markov model of fibrosis development in nonalcoholic fatty liver disease predicts fibrosis progression in clinical cohorts
**Jane Knöchel, Linnéa Bergenholm, Eman Ibrahim, Stergios Kechagias, Sara Hansson, Mathias Liljeblad, Patrik Nasr, Björn Carlsson, Mattias Ekstedt and Sebastian Ueckert**
